# A small-molecule/cytokine combination enhances hematopoietic stem cell proliferation via inhibition of cell differentiation

**DOI:** 10.1186/s13287-017-0625-z

**Published:** 2017-07-18

**Authors:** Lan Wang, Xin Guan, Huihui Wang, Bin Shen, Yu Zhang, Zhihua Ren, Yupo Ma, Xinxin Ding, Yongping Jiang

**Affiliations:** 1 Biopharmaceutical R&D Center, Chinese Academy of Medical Sciences & Peking Union Medical College, Suzhou, China; 2 Biopharmagen Corp, Suzhou, China; 3 Department of Pathology, The State University of New York at Stony Brook, Stony Brook, NY USA; 4 College of Nanoscale Science and Engineering, SUNY Polytechnic Institute, Albany, NY USA

**Keywords:** Hematopoietic stem cells, Small molecules, Expansion, Differentiation inhibition

## Abstract

**Background:**

Accumulated evidence supports the potent stimulating effects of multiple small molecules on the expansion of hematopoietic stem cells (HSCs) which are important for the therapy of various hematological disorders. Here, we report a novel, optimized formula, named the SC cocktail, which contains a combination of three such small molecules and four cytokines.

**Methods:**

Small-molecule candidates were individually screened and then combined at their optimal concentration with the presence of cytokines to achieve maximum capacity for stimulating the human CD34^+^ cell expansion ex vivo. The extent of cell expansion and the immunophenotype of expanded cells were assessed through flow cytometry. The functional preservation of HSC stemness was confirmed by additional cell and molecular assays in vitro. Subsequently, the expanded cells were transplanted into sublethally irradiated NOD/SCID mice for the assessment of human cell viability and engraftment potential in vivo. Furthermore, the expression of several genes in the cell proliferation and differentiation pathways was analyzed through quantitative polymerase chain reaction (qPCR) during the process of CD34^+^ cell expansion.

**Results:**

The SC cocktail supported the retention of the immunophenotype of hematopoietic stem/progenitor cells remarkably well, by yielding purities of 86.6 ± 11.2% for CD34^+^ cells and 76.2 ± 10.5% for CD34^+^CD38^–^ cells, respectively, for a 7-day culture. On day 7, the enhancement of expansion of CD34^+^ cells and CD34^+^CD38^–^ cells reached a maxima of 28.0 ± 5.5-fold and 27.9 ± 4.3-fold, respectively. The SC cocktail-expanded CD34^+^ cells preserved the characteristics of HSCs by effectively inhibiting their differentiation in vitro and retained the multilineage differentiation potential in primary and secondary in vivo murine xenotransplantation trials. Further gene expression analysis suggested that the small-molecule combination strengthened the ability of the cytokines to enhance the Notch pathway for the preservation of HSC stemness, and inhibited the ability of the cytokines to activate the Wnt pathway for HSC differentiation.

**Conclusions:**

We developed an optimal small-molecule/cytokine combination for the enhancement of HSC expansion via inhibition of differentiation. This approach indicates promising application for preparation of both the HSCs and the mature, functional hematopoietic cells for clinical transplantation.

**Electronic supplementary material:**

The online version of this article (doi:10.1186/s13287-017-0625-z) contains supplementary material, which is available to authorized users.

## Background

Hematopoietic stem cells (HSCs) are rare, constituting less than 0.01% of the bone marrow [1]. They have intriguing characteristics, such as self-renewal and multilineage differentiation, that are essential for the production and maintenance of all the cellular elements of a homeostatic immune and hematopoietic system [2, 3]. Ex vivo expansion is one of the viable solutions for acquiring considerable numbers of HSCs in view of their limited numbers in umbilical cord blood (UCB), poor mobilization of bone marrow stem cells, and the lack of ethnic diversity to offer adequately matched materials [3] in clinical use for various hematological disorders.

For decades, the diverse combinations of various cytokines have been extensively used to support HSC expansion in vitro, but the expansions achieved have been modest and transient [4]. The actual outcome of these efforts are truly disappointing [5] because the HSCs expanded by these protocols cannot sustain HSC self-renewal and typically undergo differentiation [6]. Subsequently, multiple other experimental approaches have been explored, including coculture with stromal cells [5, 7], ectopic expression of specific genes [1], and additions of extrinsic developmental regulators or proteins [8–10] and small-molecule chemical compounds [11–17]. Even though progress has been made by most attempts and some of the approaches have already been assessed in clinical trials, the outcomes remain unsuccessful which may be due partly to insufficient abundance of progenitor populations or improper HSC differentiation [18–20]. Nowadays, ex vivo expansion is still a critical challenge in HSC-based therapy as it requires maintenance of sustained self-renewal as well as inhibition of differentiation [5, 21].

In order to overcome this limitation, we have identified a cocktail of three small molecules and four cytokines, named as the SC cocktail. Here, we report that the SC cocktail is effective in maintaining high stemness of hematopoietic stem/progenitor cells and in promoting cell proliferation by inhibiting cell differentiation.

## Methods

### HSC isolation

Ficoll-Hypaque (GE Healthcare, Norway) density-gradient centrifugation and immunomagnetic selection (Miltenyi Biotec, Germany) were used for the isolation of CD34^+^ cell as reported previously [22]. Highly purified (>90%) CD34^+^ cells were confirmed by flow cytometry (Becton Dickinson, USA).

### Small-molecule selection

Peripheral blood (PB) CD34^+^ cells were cultured in the serum-free Stemspan SFEM medium (Stem Cell Technologies, Canada) supplemented with three basic cytokines: 40 ng/mL thrombopoietin (TPO), 100 ng/mL stem cell factor (SCF), and 100 ng/mL Fms-related tyrosine kinase 3 ligand (Flt3-L; Biopharmagen Corp., China); five compounds named stemregenin1 (SR1), Scriptaid (SCR; Selleck Chemicals, USA), CAY10633 (C633), CAY10433 (C433; Cayman Chemical, USA), and valproic acid (VPA; Sigma-Aldrich, USA) at various concentrations [14, 16, 23] (Additional file 1: Table S1) were added at the same time. Dimethyl sulfoxide (DMSO) plus cytokines served as a vehicle control, and cytokines only as a negative control. Cell viability and apoptosis were detected using CCK-8 (Dojindo, Japan) and PE Annexin V Apoptosis Detection Kit I (Becton Dickinson) according to the technical manual. The cells were maintained at 37 °C in a humidified atmosphere containing 5% of CO_2_ for 7 days. The selected compounds were then combined at their selected optimal concentration for 12 days (Additional file 2: Table S2a). Cell numbers and marker expression were determined and analyzed on days 7, 9, and 12. PB CD34^+^ cells were then cultured with the basal medium supplemented with three cytokines and 1 μM SR1; various concentrations of VPA or C433 were added separately to the culture system. A further modulation of C433 was conducted to obtain the final optimal small-molecule combination (Additional file 2: Table S2b–d).

### Cytokine combination optimization

Cord blood CD34^+^ cell confirmation was first established in a 24-well plate for 7 days. Considering their significantly more prominent expression of markers of early progenitors [24] and widespread application [25–27], we used UCB cells in our further analyses instead of PB cells. For optimization of the cytokine combination, culturing was still performed in 24-well plates; 20 ng/mL interleukin (IL)-3 and 50 ng/mL IL-6 (Biopharmagen Corp.) were added separately or in combination, besides the three basic cytokines and the three selected small molecules, in order to augment the yield of cells (Additional file 3: Table S3).

### Cell counting and phenotypic analysis

Viable cells were enumerated by the trypan blue exclusion method and cellular expansion fold was calculated based on the initial inputs. Cells were collected and stained with an anti-human CD34 monoclonal antibody conjugated to phycoerythrin (PE; Miltenyi) and an anti-human CD38 monoclonal antibody conjugated to allophycocyanin (APC; Becton Dickinson), together or separately, or their related isotype control antibodies. Flow cytometry was performed and data was analyzed using a BD FACS Verse system (Becton Dickinson).

### Cell cycle analysis

The cell cycle status of uncultured cord blood HSCs (PC), SC cocktail-treated cells (SC cocktail), and vehicle controls (VC) were assessed by means of the CycleTEST™ plus DNA Reagent Kit (Becton Dickson) according to the manufacturer’s instructions. Briefly, 10^6^ day-7 cells were harvested and washed twice with phosphate-buffered saline (PBS). Then, they were fixed overnight with 4% paraformaldehyde at 4 °C before staining. Solutions A and B were added successively and were allowed to act for 10 min at room temperature after the centrifugation of the fixed cells. After that, solution C was added and incubated for 10 min at 4 °C in the dark. The samples were analyzed on the FACS Verse flow cytometer (Becton Dickson); data analysis was performed in Flowjo software (Tree Star Inc, USA).

### Colony-forming assay

Colony-forming units (CFUs) were generated by seeding cells into cytokine-containing methylcellulose media (Methocult C H4034 optimal; Stem Cell Technologies), and 40 ng/mL TPO was supplied to the media for the detection of CFUs-megakaryocyte (CFUs-Mk). The colonies including burst-forming units-erythroid (BFUs-E), CFUs-granulocyte, macrophage (CFUs-GM), CFUs-granulocyte, erythrocyte, macrophage, megakaryocyte (CFUs-GEMM), and CFUs-Mk were scored on day 14 under an inverted microscope (Olympus Corporation, Japan).

### RNA extraction and qPCR

Total RNA was extracted using RNAiso Plus (TAKARA, China). Equivalent amounts of cDNA was used as a template for the polymerase chain reaction (PCR), and the cDNA was reverse-transcribed by means of the RevertAid First Strand cDNA Synthesis Kit (Thermo Fisher, USA); reverse-transcription PCR (RT-PCR) was run using the Phusion High-Fidelity DNA polymerase (NEB, USA) on the Veriti 96-Well Thermal Cycler (Applied Biosystems, USA) and β*-actin* served as an internal standard. The amplicons were analyzed on a 3% agarose gel (TAKARA) stained with ethidium bromide (Thermo Fisher) with a 500-bp ladder of DNA markers (TAKARA). Quantitative PCR (qPCR) was performed with the Power SYBR Green PCR Master Mix (Thermo Fisher) on the Step-One Plus real-time PCR system (Applied Biosystems) and all experiments were conducted in triplicate. The quantitation of each gene was performed by using SYBR Green qPCR. The *HPRT1*-normalized transcript data are shown as relative expression levels in the SC cocktail and VC groups compared to the corresponding level in primary uncultured cord blood CD34^+^ cells. A non-cDNA template was included in each assay as a negative control. The primer sequences are listed in Additional file 4: (Table S4).

### Animals and xenotransplantation assays

Nonobese diabetic/severe combined immunodeficient (NOD/SCID) mice (specific pathogen-free; 6–8 weeks old, weight 16.0–17.6 g, male), obtained from SLAC Laboratory Animal Co. (Shanghai, China), were used for CD34^+^ cell transplantation. For each group, 5 × 10^5^ cells were injected intravenously via the tail into sublethally irradiated (X-ray 2.5 Gy, Pxi X-RAD 320IX, Australia) NOD/SCID mice 4 h after irradiation. Human CD45^+^, CD34^+^, CD34^+^CD38^–^ (a marker of more primitive hematopoietic progenitors), and other blood cell lineages, including myeloid (CD14), lymphoid (CD19), erythroid (CD71), neutrophil (CD66), and megakaryocyte (CD41a), were monitored at week 8 by flow cytometric analysis of bone marrow cells acquired from femurs and tibiae and PB cells obtained from the retro-orbital vein.

From each primary recipient mouse, 50% of the harvested bone marrow cells were transplanted into a second sublethally irradiated NOD/SCID mouse for the secondary transplantation assessment. At 10 weeks post-engraftment, the percentage of human CD45-FITC, CD34-APC or -PE, CD38-APC, CD19-APC, CD14-PE, CD71-APC, CD66-FITC, and CD41a-APC cells (all antibodies from Becton Dickson) among bone marrow cells were analyzed by flow cytometry. Ten mice were used for each transplantation group, and five additional mice received saline after irradiation as a negative control which was used for subtracting the background signal.

### Statistical analysis

Results are presented as means ± SD from varying numbers of independent experiments. Differences were evaluated by Student’s two-tailed *t* test or one-way analysis of variance (ANOVA) with pairwise comparison for equal variance, as specified. Differences are designated to be significant when *p* < 0.05.

## Results

### Selections of small molecules for HSC expansion while maintaining the CD34^+^ immunophenotype

**Fig. 1:**
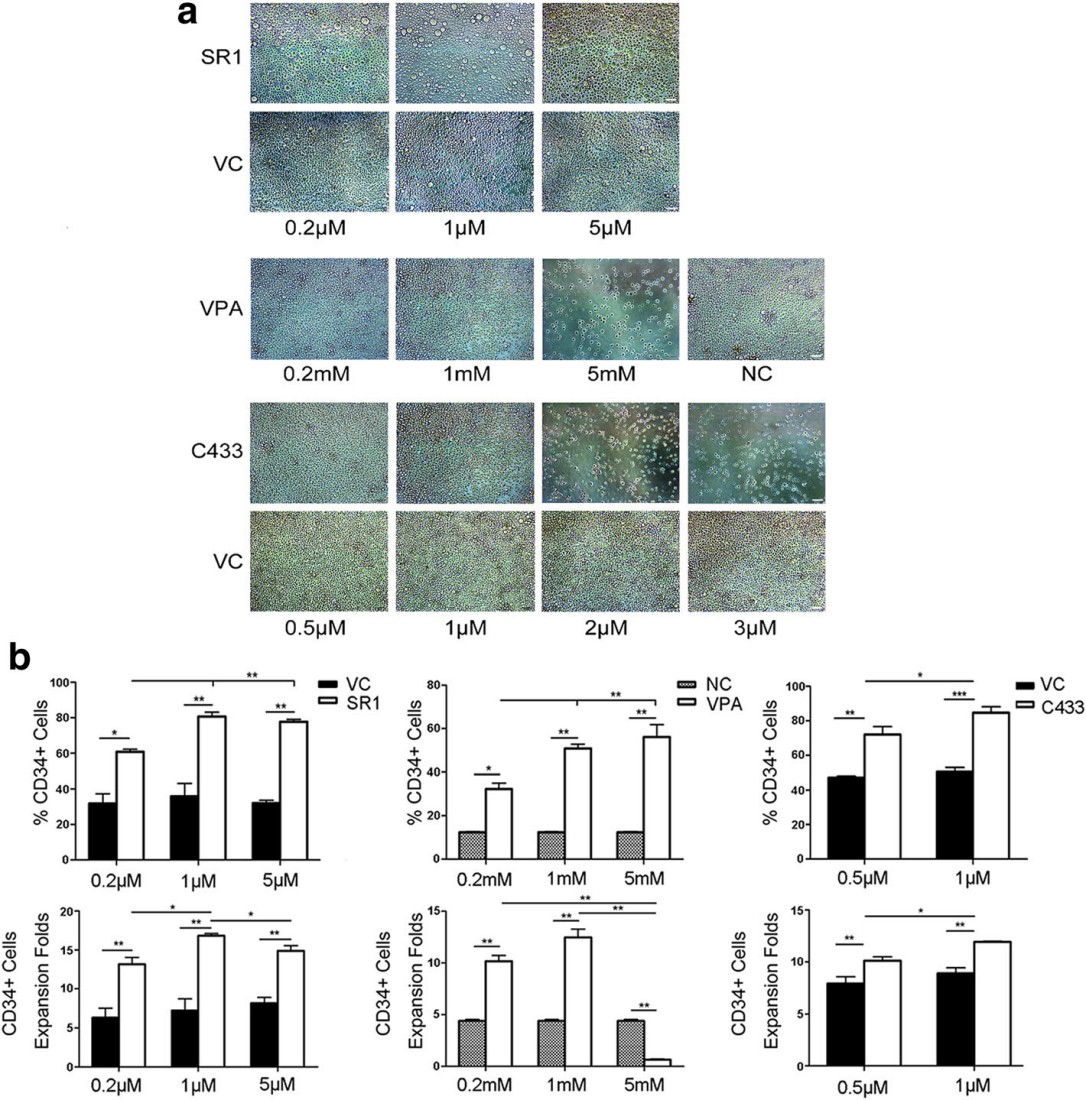
Effects of small molecules on cell morphology and expansion. **a** Cell morphology for cultures with various small molecules at three different concentrations. Photos were captured on day 7 (20× objective, *scale bar* = 50 μm). The vehicle control (*VC*) was composed of cytokines and DMSO; the negative control (*NC*) consisted of cytokines only. **b** CD34-positive rate and fold expansion of CD34^+^ cells for the different concentrations of each single small molecule on day 7. Data are shown as mean ± SD, *n* = 3. *p < 0.05, **p < 0.01, ***p < 0.001. *C433* CAY10433, *SR1* stemregenin1, *VPA* valproic acid

According to the morphology, apoptosis and decreased cell numbers were observed when the concentrations of VPA and C433 were above 1 mM and 1 μM, respectively; SR1 at 0.2 μM and 1 μM yielded an excellent growth state, but was relatively less beneficial when its concentration was at 5 μM (Fig. 1a and Additional file 5: Figure S1). Among the five candidates, Scriptaid (SCR) and CAY10633 (C633) were excluded based on their unsatisfying effects on immunophenotype maintenance. SR1 at 1 μM showed an optimal result for the CD34-positive rate and cell proliferation during the 7-day culturing. VPA at 1 mM benefited both the marker expression and cellular fold expansion. C433 at 1 μM not only maintained high CD34^+^ purity, but also caused a greater fold expansion of CD34^+^ cells (Fig. 1b).

### Effects of small-molecule combinations on HSC proliferation and differentiation

**Fig. 2:**
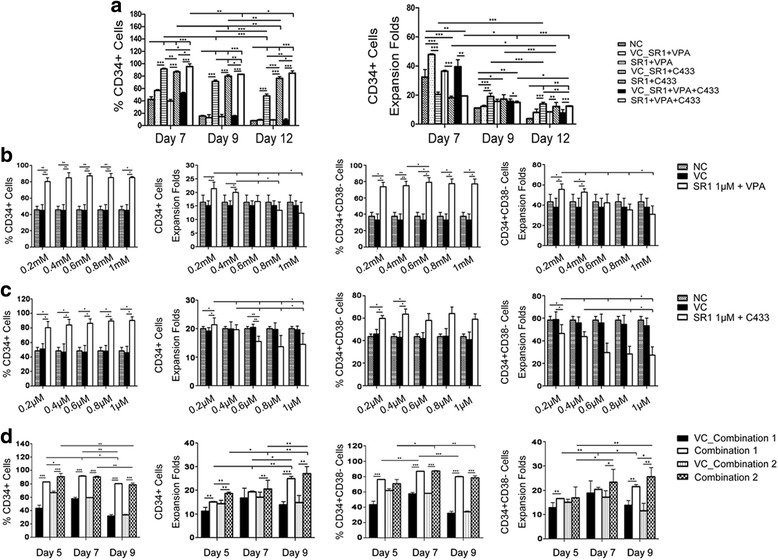
Effects of small-molecule combinations on marker purity and cellular fold expansion. **a** CD34 expression and CD34^+^ cellular fold expansion for different combinations of small molecules. **b**,**c** VPA and C433 concentration adjustment. PB CD34^+^ cells were cultured with the three core cytokines, 1 μM stemregenin1 (*SR1*), and various concentrations of valproic acid (*VPA*) (**b**) or CAY10433 (*C433*) (**c**). **d** Final optimization of the compound combination. Data are shown as mean ± SD, *n* = 3. *p < 0.05, **p < 0.01, ***p < 0.001. *NC* negative control, *VC* vehicle control

For the purpose of obtaining the most effective small-molecule combination for HSC proliferation, the three selected compounds, coupled with three basic cytokines, were mixed in different combinations at their individually identified optimal concentrations. A stably enhanced CD34-positive rate could be maintained by a combination of SR1, VPA, and C433 throughout the 12 days of culturing, reaching the highest proportion on day 7 (Fig. 2a and Table 1). Owing to strong cytotoxicity, insufficient numbers of cells survived in the VPA + C433 group; besides, the expansion of cells was inadequate without the support of cytokines as shown in the compounds-only and basal medium-only groups (Table 1 and Additional file 6: Figure S2). A combination consisting of SR1 and VPA showed a time-dependent decrease in CD34 expression, and CD34^+^ purity of the SR1 + C433 group ranged from 86% to 76% throughout the culture period (Fig. 2a and Table 1). The fold expansion of CD34^+^ cells, however, showed no advantage for the three combinations (Fig. 2a). It appeared that the negative effects of the individual small molecules were additive when we used them together at the individually optimized concentrations.

**Table 1:** CD34 expression on day 7 after treatment with different small-molecule combinations

Groups	Medium content		CD34 (%)
	Small molecules	Cytokines	
SMC	SR1 + VPA	TSF	90.5 ± 0.5*^#†‡^
	SR1 + C433		86.6 ± 12.5*^#§^
	SR1 + VPA + C433		94.5 ± 4.3*^#^
VC	DMSO	TSF	56.6 ± 1.8
			39.8 ± 0.7
			52.2 ± 1.7
SMCO	SR1 + VPA	Absent	N/A
	SR1 + C433		
	SR1 + VPA + C433		
NC	Absent	TSF	45.5 ± 0.2
BC	Absent	Absent	N/A

The cells were not enough for the flow cytometry detection of the SMCO group

Data are shown as mean ± SD (*n* = 3)

*p < 0.01, SMC vs VC; ^#^ *p* < 0.01, SMC vs NC; ^†^ *p* < 0.01, SR1 + VPA vs SR1 + C433; ^‡^ *p* < 0.05, SR1 + VPA vs SR1 + VPA + C433; ^§^ *p* < 0.05, SR1 + C433 vs SR1 + VPA + C433

*BC* basal control, *C433* CAY10433, *DMSO* dimethyl sulfoxide, *N/A* not applicable, *NC* negative control, *SMC* small-molecule combination, *SMCO* small-molecule combination only, *SR1* stemregenin1, *TSF* TPO + SCF + Flt3-L, *VC* vehicle control, *VPA* valproic acid

In order to resolve this issue, a modulation was performed for VPA and C433. During the selection of VPA, the percentage of CD34^+^ was between 80% and 85% and CD34^+^CD38^–^ purity was ~80% without a statistically significant difference among the five concentrations, except between 0.4 mM and 0.6 mM for the CD34^+^CD38^–^ proportion. A dose-dependent effect was observed for the fold expansion of cells, with the highest fold change at 0.2 mM (Fig. 2b). For the C433 adjustment, the CD34-positive rate was maintained above 80% with an increased dose-related effect and the percentage of CD34^+^CD38^–^ cells was ~60%. In contrast to the immunophenotype, however, the cellular fold expansion for the two kinds of cells showed a reverse pattern. Although C433 had no positive effect on the expansion of CD34^+^CD38^–^ cells, its 0.2 μM concentration not only yielded a higher CD34^+^ cellular fold expansion, but also maintained 80.6% ± 8.2% CD34^+^ proportion and 60.7% ± 2.7% CD34^+^CD38^–^ proportion (Fig. 2c). These data indicated that C433 was more effective at preserving the HSC immunophenotype rather than promoting proliferation.

Further selection of the C433 concentration was performed, considering its comparatively high toxicity and in order to obtain the minimal effective concentration in the combination. On day 7, both the CD34^+^ and CD34^+^CD38^–^ proportions were held above 85% for the two combinations with no significant difference. A time-related ascendant tendency was observed for the cell expansion of the two combinations. Combination 1 showed no statistical significance for the cell proliferation compared to vehicle control on day 7, whereas combination 2 had the advantage of cell proliferation both on day 7 and day 9 (Fig. 2d). To sum up, these data implied that C433 at 0.1 μM was no different from 0.2 μM in the compound mixture in terms of benefits for both the HSC phenotype and cellular fold expansion; the suitable time point, day 7, was confirmed as well—it showed much higher specific marker expression and cell proliferation efficiency.

### Optimizing the cytokine combination supplemented with selected small molecules for the expansion of HSCs

**Fig. 3:**
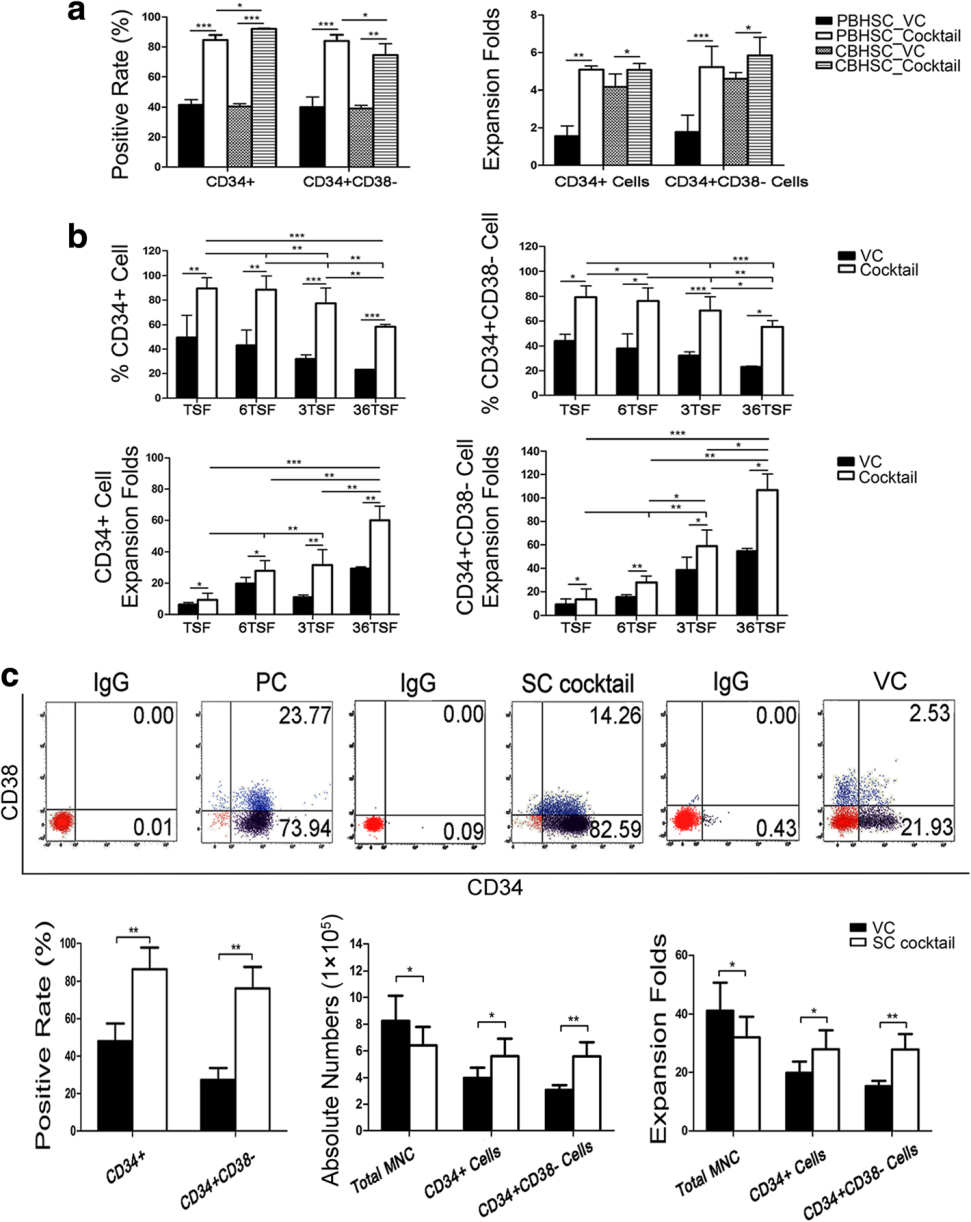
Effects of optimal cytokine combination supplemented with selected small molecules on cell expansion. **a** Confirmation of PB and cord blood CD34^+^ cells cultured in a 24-well plate. **b** Optimal cytokine combination screening. Cord blood CD34^+^ cells were primed in the presence or absence of the small-molecule combination supplemented with different groups of cytokines for 7 days. **c** Representative flow cytometry results of CD34 and CD38 percentage, and phenotypic changes and cellular fold expansion caused by the optimal formula for day 7. All data are shown as means ± SD, *n* = 3. *p < 0.05, **p < 0.01, ***p < 0.001. *CBHSC* cord blood hematopoietic stem cell, *IgG* immunoglobulin G, *MNC* mononuclear cells, *PBHSC* peripheral blood hematopoietic stem cell, *PC* uncultured CBHSCs, *TSF* TPO + SCF + Flt-3 L, *3TSF* IL-3 + TPO + SCF + Flt-3 L, *6TSF* IL-6 + TPO + SCF + Flt-3 L, *36TSF* IL-6 + IL-3 + TPO + SCF + Flt-3 L, VC vehicle control

To verify the effect of a selected small-molecule combination on cord blood CD34^+^ cells in a larger culture system, we then performed confirmation tests for both PB and cord blood CD34^+^ cells in 24-well plates. The percentages of CD34^+^ and CD34^+^CD38^–^ were consistent with the aforementioned findings and were ~85% for both CD34^+^ and CD34^+^CD38^–^ in expanded PB CD34^+^ cells; and 92.0% ± 0.5% for CD34^+^ and 74.6% ± 7.6% for CD34^+^CD38^–^ in expanded cord blood CD34^+^ cells. However, there was only approximately five-fold expansion of CD34^+^ cells and CD34^+^CD38^–^ cells among PB and cord blood CD34^+^ cells (Fig. 3a). Therefore, a cytokine screening was subsequently performed to enhance the yield of cells.

Four groups were tested; they contained the optimized small-molecule combination and distinct cytokines named TPO + SCF + Flt3-L (TSF), IL-6 + TPO + SCF + Flt3-L (6TSF), IL-3 + TPO + SCF + Flt3-L (3TSF), and IL-3 + IL-6 + TPO + SCF + Flt3-L (36TSF). The attenuated HSC phenotype correlated with strengthened cell proliferation. The use of IL-3 was helpful for the cell expansion, but was accompanied with a markedly decreased marker purity compared to the groups without IL-3 (Fig. 3b and Table 2).

**Table 2:** Surface marker expression on day 7 after treatment with a cytokine combination

Groups	Medium content		CD34^+^ (%)	CD34^+^CD38^–^ (%)
	Cytokines	Compounds		
Cocktail	TSF	SR1 + VPA + C433	89.6 ± 8.6 ^A,B,C,D^	79.6 ± 8.9 ^a, b, c, d^
	6TSF		86.6 ± 11.2 ^A,E,F^	76.2 ± 10.5 ^a, e, f^
	3TSF		77.3 ± 10.8 ^A,G^	68.5 ± 11.2 ^a, g^
	36TSF		58.1 ± 2.1 ^A^	55.6 ± 4.9 ^a^
VC	TSF	DMSO	49.4 ± 18.2	44.1 ± 5.3
	6TSF		43.0 ± 12.5	38.0 ± 11.9
	3TSF		32.2 ± 3.2	32.2 ± 3.2
	36TSF		23.1 ± 0.5	21.1 ± 0.5

Data are shown as mean ± SD (*n* = 3)

^A^ *p* < 0.01, cocktail vs VC; ^B^ *p* < 0.05, TSF vs 6TSF; ^C^ *p* < 0.01, TSF vs 3TSF; ^D^ *p* < 0.001, TSF vs 36TSF; ^E^ *p* < 0.01, 6TSF vs 3TSF; ^F^ *p* < 0.01, 6TSF vs 36TSF; ^G^ *p* < 0.01, 3TSF vs 36TSF. For CD34^+^CD38^–^ rate analysis, ^a^ *p* < 0.01, cocktail vs VC; ^b^ *p* < 0.05, TSF vs 6TSF; ^c^ *p* < 0.001, TSF vs 3TSF; ^d^ *p* < 0.001, TSF vs 36TSF; ^e^ *p* < 0.01, 6TSF vs 3TSF; ^f^ *p* < 0.01, 6TSF vs 36TSF; ^g^ *p* < 0.05, 3TSF vs 36TSF

*C433* CAY10433, *DMSO* dimethyl sulfoxide, *SR1* stemregenin1, *TSF* TPO + SCF + Flt-3 L, *3TSF* IL-3 + TPO + SCF + Flt-3 L, *6TSF* IL-6 + TPO + SCF + Flt-3 L, *36TSF* IL-6 + IL-3 + TPO + SCF + Flt-3 L, *VC* vehicle control, *VPA* valproic acid

Considering the fold expansion and HSC-specific phenotype, 6TSF together with the selected small-molecule combination resulted in higher CD34^+^ and CD34^+^CD38^–^ percentages, as well as greater cellular fold expansion. Thus, the optimal formula, named as the SC cocktail, consisting of four cytokines (TPO, SCF, Flt3-L, and IL-6) and three small molecules (SR1, VPA, and C433), was finally obtained, which achieved the maximum stimulation of the CD34^+^ and CD34^+^CD38^–^ cell expansion (28.0 ± 5.5- and 27.9 ± 4.3-fold expansion, respectively; Fig. 3c) while providing adequate inhibition of cell differentiation (CD34^+^ 86.6% ± 11.2% and CD34^+^CD38^–^ 76.2% ± 10.5%; Fig. 3c) ex vivo.

### Ability of the optimal SC cocktail to induce HSC expansion while sustaining stemness in vitro

**Fig. 4:**
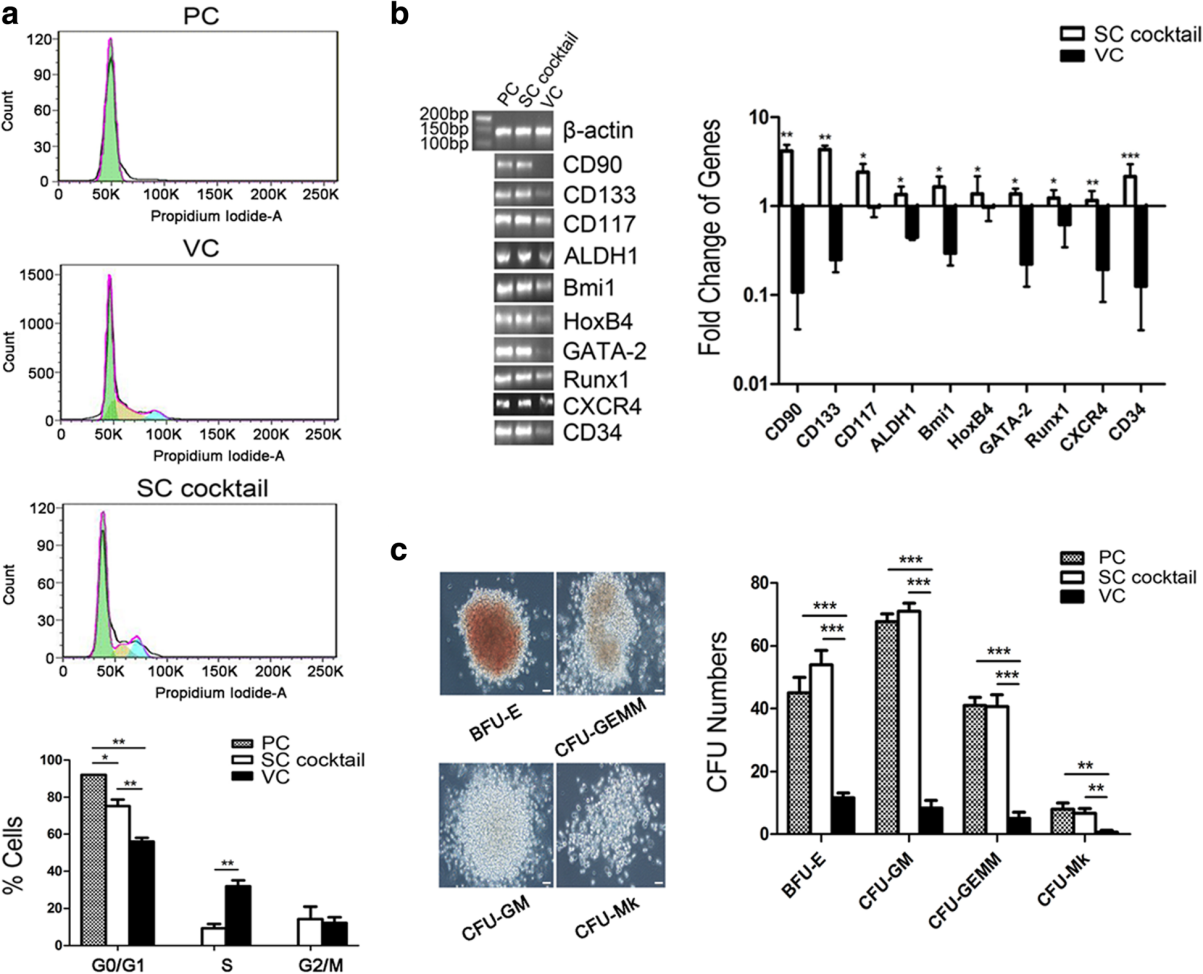
In vitro functional assessments of the SC cocktail-expanded CD34^+^ cells. **a** Cell cycle analysis. One of three representative experiments is shown, and the percentage of different phases is summarized in the histogram. **b** HSC-specific gene expression. Results of qualitative RNA-PCR are shown on the left for one representative of three independent RNA samples analyzed per group, and the results of quantitative PCR are shown in the right. **c** The morphology and numbers of colony-forming units (*CFUs*). Morphology (20× objective, *scale bar* = 50 μm) and colony numbers were recorded on day 14 after cell seeding. All data are shown as means ± SD, *n* = 3. *p < 0.05, **p < 0.01, ***p < 0.001. *BFU-E* burst-forming unit-erythroid, *CFU-GEMM* CFU-granulocyte, erythrocyte, macrophage, megakaryocyte, *CFU-GM* CFU-granulocyte, macrophage, *CFU-Mk* CFU-megakaryocyte, *PC* uncultured cord blood HSCs, *VC* vehicle control

We next characterized these expanded cells after treatment with the SC cocktail in vitro. Cell cycle results showed that the PC group was relatively more quiescent and mainly stayed at the G0/G1 phase (92.0% ± 0.1%), in accord with the features of HSCs; most of the SC cocktail group, just as the PC group, resided at the G0/G1 (75.2% ± 3.6%) phase with a lower S phase proportion (9.2% ± 2.4%) compared to the VC group; the proportion of G0/G1 cells for the VC group on the other hand was much lower and there was active DNA synthesis (G0/G1: 56.0% ± 2.0%, S: 31.8% ± 3.2%) after 7 days of culturing (Fig. 4a). It has been posited that a rapid cell cycle and cell division are responsible for the loss of HSC properties [16] and directly leads to the production of mature blood cells; therefore, these data indicate that cells treated with the small-molecule combination in the SC cocktail do not show enhanced differentiation. In contrast, cells treated without the small-molecule combination exhibited a trend of enhanced differentiation.

Subsequently, RT-qPCR was performed in order to determine the expression of typical genes involved in HSC stemness. Based on the RT-PCR results, it was found that HSC-specific markers and functionally important genes (*CD90*, *CD133*, *CD117*, *ALDH1*), and other HSC-relevant genes and transcriptional factors (*Bmi1*, *HoxB4*, *GATA-2*, *Runx1*, and *CXCR4*), are all expressed in both the PC and SC cocktail groups. According to the qPCR results, all those genes were upregulated in the SC cocktail group after normalization to the level of the PC group (Fig. 4b). In contrast, the expression of these genes was dramatically reduced or barely detectable in the VC group. Given that those genes are pivotal for HSC self-renewal, bone marrow colonization, progenitor pool preservation, and HSC differentiation restraint [21, 28, 29], these data confirmed that the SC cocktail-generated CD34-positive cells still preserved and enhanced their HSC properties. In contrast, cells treated without the compounds exhibited an enhanced differentiation tendency.

Finally, a CFU assay was performed to determine whether a single newly formed cell could randomly commit to multilineage hematopoietic progenitors. The results showed that the SC cocktail-expanded CD34^+^ cells behaved the same as the untreated HSCs and were giving rise to diverse types of myeloid colonies, including 54.0 ± 4.6 BFUs-E, and multipotential progenitors, such as 71.0 ± 2.7 CFUs-GM, 40.7 ± 3.8 CFUs-GEMM, and 6.7 ± 1.5 CFUs-Mk on day 14 (Fig. 4c). These data showed that the potential of multilineage differentiation was preserved in SC cocktail-treated CD34^+^ cells in vitro.

### Ability of the optimal SC cocktail to enhance the engraftment potential in vivo

**Fig. 5:**
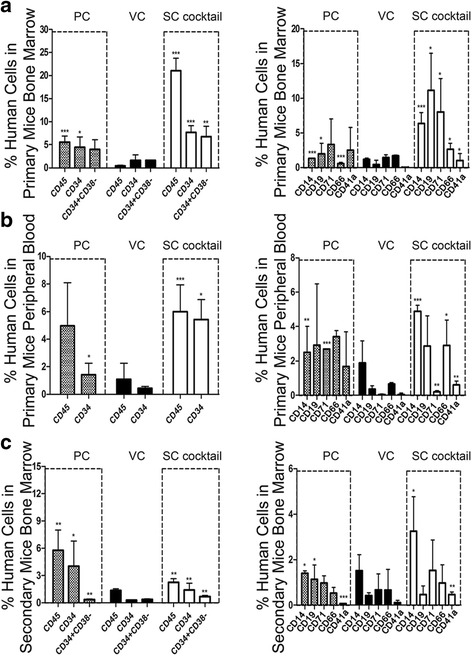
Functional assessment of the SC cocktail-expanded CD34+ cells in NOD/SCID mice. **a** The presence of human cells in bone marrow 8 weeks after primary transplantation. **b** The presence of human cells in peripheral blood 8 weeks after primary transplantation. **c** The presence of human cells in bone marrow 10 weeks after secondary transplantation. Ten mice were used for each group and five additional mice receiving saline were used to subtract the background signal. Data are shown as means ± SD. *p < 0.05, **p < 0.01, ***p < 0.001. *PC* uncultured cord blood HSCs, *VC* vehicle control

Besides the in vitro confirmation, we then transplanted these expanded cells into NOD/SCID mice to evaluate their in vivo functional capability. Various humanized hematopoietic cells were detectable in the bone marrow of all recipients 8 weeks after engraftment. Human CD45^+^ cells (hCD45^+^) remained in greater numbers in the PC group than in the VC group; similar group differences were also found for the CD34^+^ and CD34^+^CD38^–^ cells. In contrast, the hCD45^+^ chimerism in the SC cocktail group was distinctly increased, as was CD34^+^ and CD34^+^CD38^–^ purity. Meanwhile, the capacity for most types of multilineage differentiation in the SC cocktail group was markedly different from that in the PC and VC groups, with advantages in myeloid CD14^+^, CD19^+^, and CD71^+^ (Fig. 5a). In peripheral blood, the abundance of hCD45^+^ and hCD34^+^ cells was high in the PC group, but it was much better in the SC cocktail group, compared to those achieved in the VC group; some other mature hematopoietic cells were released into the peripheral blood and most of them also showed definite superiority in the SC cocktail group (Fig. 5b). These primary transplantation data confirmed the enhanced multilineage differentiation capacity of SC cocktail-generated CD34^+^ cells in vivo.

Furthermore, the potential for self-renewal was estimated by secondary transplantation. In the bone marrow of secondary recipients 10 weeks after engraftment, even though the presence of hCD45^+^ cells and hCD34^+^ in the SC cocktail group was not as high as in the PC group, the proportion of CD34^+^CD38^–^, CD14^+^, CD19^+^, and CD41a^+^ were notably upregulated as compared to the PC and VC groups (Fig. 5c). These data confirmed that the SC cocktail might improve the capacity for long-term engraftment and multilineage differentiation in an in vivo environment and indicated a powerful potential for self-renewal. Moreover, during the primary and secondary transplantation, it is important to note that all the recipients continued to live without apparent abnormalities. This observation validated the in vivo long-term safety of our SC cocktail-expanded cells.

### Ability of the optimal SC cocktail to modulate the Notch and Wnt signaling pathways

**Fig. 6:**
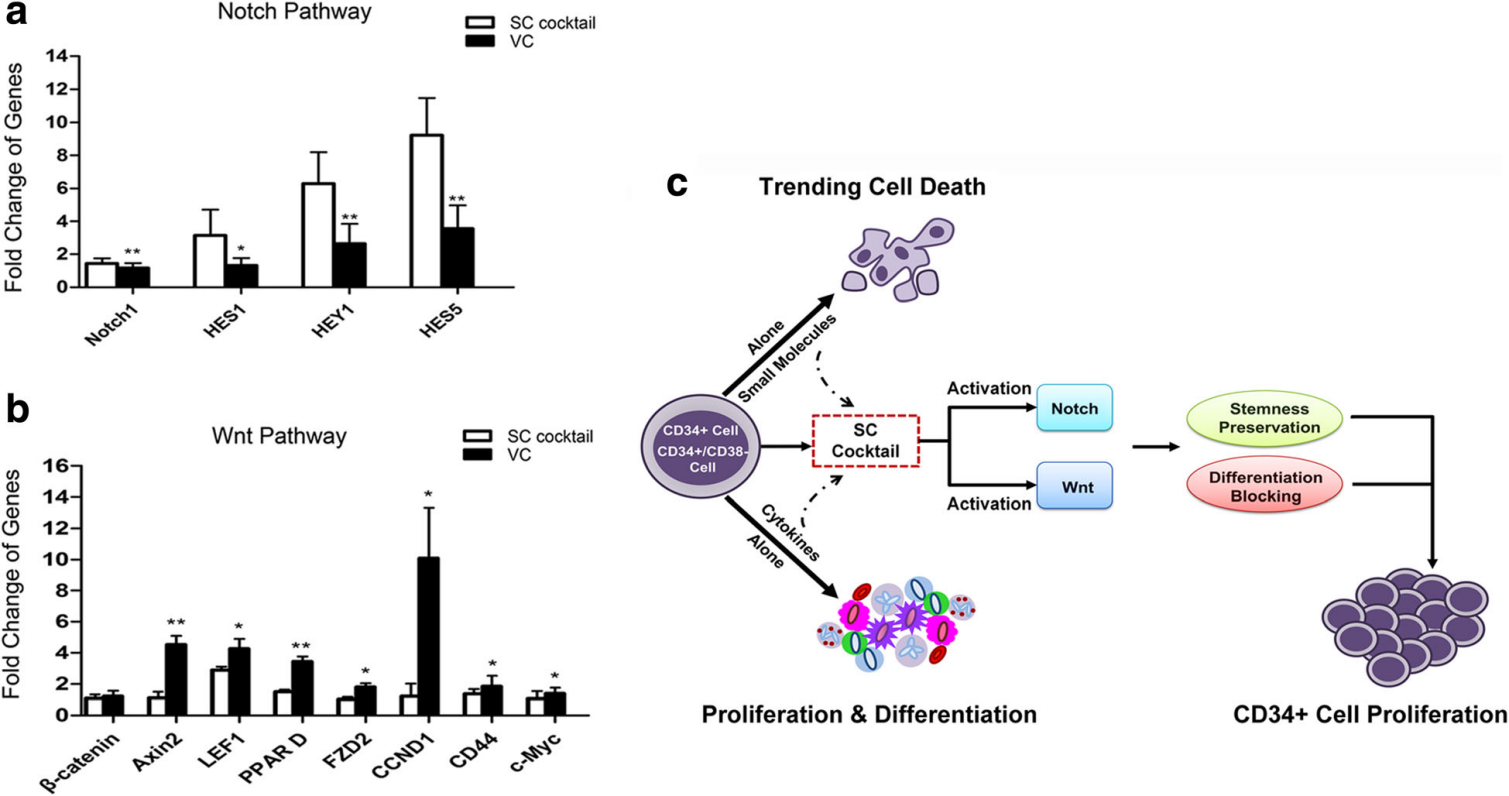
Effects of the small-molecule combination on the expression of Notch and Wnt signaling genes in human cord blood CD34^+^ cells. **a** The relative expression of Notch pathway key genes and target genes. **b** The relative expression of Wnt pathway key genes and target genes. Data are shown as mean ± SD, *n* = 3. *p < 0.05, **p < 0.01, ***p < 0.001. **c** A schematic summary of the effects of the SC cocktail on CD34^+^ cell proliferation and differentiation. *VC* vehicle control

Given that the small molecules preserved the HSC phenotype with a blockage of differentiation rather than promotion of the expansion of total mononuclear cells, we then investigated the expression by qPCR of target genes for two major signaling pathways related to HSC properties and differentiation, Notch and canonical Wnt [30–34], to reveal the potential mechanisms. The results indicated that the relative expression of the key Notch pathway gene *Notch1* was elevated in small molecule-treated cells compared to the untreated ones, and the Notch target genes *HES1*, *HEY1*, and *HES5* were also statistically significantly upregulated in the presence of the combination of compounds (Fig. 6a). The relative expression of the canonical Wnt-related key gene β*-catenin* showed no dramatic difference between the groups treated and not treated with the compounds; however, the Wnt target genes *Axin2*, *LEF1*, *PPAR D*, *FZD2*, *Cyclin D1*, *CD44*, and *c-Myc* were prominently enhanced in the compound-untreated cells as compared with the compound-induced ones (Fig. 6b). Taken together, we hypothesized that when CD34^+^ or CD34^+^CD38^–^ cells are treated with the SC cocktail, the small molecules may affect signaling of the cytokines in ways that activate the Notch and Wnt pathway for the preservation of HSC stemness, while preventing the effect of cytokines on cell differentiation, ultimately resulting in enhanced CD34^+^ cell expansion (Fig. 6c).

## Discussion

Extrinsic modulation of HSC fate is more practical for clinical application than in vivo approaches that use viral vectors. Among the multiple extrinsic regulators, the chemically well-defined small molecules can be used to regulate the intracellular procedures in a rapid, competitive, and reversible fashion through fine-tuning of their concentration and because of a lack of immunogenicity [35–39], which is the reason why we chose this strategy for the induction of ex vivo expansion of CD34^+^ cells. As demonstrated by accumulated evidence, two categories of compounds, aryl hydrocarbon receptor (AHR) antagonists and histone deacetylase inhibitors (HDACi), have had some striking success in terms of HSC expansion [14, 16, 40, 41]. AHR antagonists can directly bind and restrain AHR, which is partially responsible for HSC quiescence maintenance [42, 43], to induce the expansion of CD34^+^ cells [14]; an HDACi, on the other hand, promotes HSC proliferation through the regulation of epigenetic plasticity and chromatin structures which are critical for the maintenance of the primitive status of HSCs [16, 40, 44–46]. For the purpose of integrating the advantages of these two categories and to maximize their impact on HSC proliferation, we therefore focused on the screening of combinations of these compounds. During this process, as additive negative effects were observed when the selected compounds were combined at their individually determined optimal concentrations, a component adjustment had to be undertaken for the augmentation of the cell yield. SR1 showed lower cytotoxicity than the others, and our previous work also confirmed its capability at 1 μM for better maintenance of CD34^+^ cells; hence, only two components were adjusted in the compound combination. After multiple rounds of selection, the synergistic effect of these small molecules, coupled with three cytokines, was ultimately acquired which benefits the blocking of HSC differentiation, with a CD34-positive rate of about 92.0% ± 0.5% and primitive-progenitor proportion (CD34^+^CD38^–^) of about 74.6% ± 7.6%, instead of promoting the total mononuclear cell proliferation.

Unlike previously reports [16], the role of cytokines is irreplaceable and necessary for HSC culture. We found that there were not enough cells left or cell trending death on day 7 in the absence of cytokines during small-molecule combination screening. In addition, TPO, SCF, and Flt3-L have long been confirmed as the core cytokine mix for the best support of HSCs in in-vitro culture [47–52]; therefore, they were consequently required for the small-molecule combination screening in the first place. Unexpectedly, we found that the three essential cytokines only expanded a limited number of HSCs whereas the addition of IL-6 and IL-3 significantly strengthened the output. Cells showed robust proliferation in spite of the IL-3 presence; HSC features and signals of function-relevant specific surface markers were lost and might be accompanied by differentiation, and result in the loss of HSC activity [14, 53]. As a result, IL-6 along with the three basic cytokines was identified as the optimal cytokine combination instead of IL-3. Cord blood CD34^+^ cells cultured in a medium containing these four cytokines plus the small-molecule combination for 7 days could finally reach ~28.0-fold expansion of CD34^+^ and CD34^+^CD38^–^ cells with CD34^+^ purity of 86.6% ± 11.2% and CD34^+^CD38^–^ proportion of 76.2% ± 10.5%, respectively. Our SC cocktail produced much higher CD34^+^ expansion folds with higher purity compared with the previously published work with SR1 alone (coupled with cytokines), which reached a 24.2-fold increase in CD34^+^ cells with a CD34 percentage of 42% ± 1.8% on day 7 [14], and with VPA alone (plus cytokines), which maintained CD34 purity of about 80.0% on day 7 [16]. In summary, these data demonstrated that the capacity for cell proliferation is dramatically influenced by the distinct combination of cytokines and an ideal yield could be obtained by means of the optimal cytokines. Certainly, when referring to the increase in absolute numbers of stem cells, the involvement of a limiting dilution assay can provide more convincing data [54–56] by quantitation of SCID-repopulating cells (SRCs) in a given sample before and after in vivo transplantation. In our case, only the absolute number of in-vitro generated cells was calculated; however, we believed that the apparent advantages of the SC cocktail in increasing cell expansion, blocking cell differentiation, and maintaining HSC stemness may benefit the frequency of SRCs in the immunodeficient mouse as well. This experiment will no doubt be evaluated in our future study.

Moreover, in the course of our study, we observed that HSCs cultured with the SC cocktail showed a superb undifferentiated state, affirmed by our functionality studies including cell cycle analysis, HSC-specific gene expression, the CFU assay in vitro, and mouse engraftment in vivo; but they did not notably increase the number of total mononuclear cells. We assumed that the inhibition of HSC differentiation might directly rely on the small-molecule combination. This assumption was further confirmed by the signaling pathway analysis. We observed an ascendant tendency for the Notch target genes in the presence of the compound combination. The upregulation of Notch pathway target genes in the SC cocktail group suggested that this pathway might be activated by the small molecules, and the downstream effects of Notch pathway activation were related to the preservation of HSC properties [31, 32, 57, 58]. Simultaneously, these data implied a stronger proliferation and differentiation ability of cells not treated with the small-molecule cocktail. Besides, based on our results from analysis of the Wnt pathway, we found that the cell differentiation was blocked due to the presence of small molecules. Other research groups, Luis et al. [34] and Famili et al. [33], also found that the canonical Wnt pathway regulates hematopoiesis in a dosage-dependent fashion. Only mildly activated Wnt signaling pathway could enhance the function of HSCs, and a high activated level of the Wnt pathway could enhance the HSC differentiation and impair HSC self-renewal. As shown in our data (Fig. 6b), the Wnt pathway was more increased in the absence of the small-molecule group (VC). On the other hand, the presence of small molecules (SC cocktail) could reduce the HSC stimulation caused by cytokines and only led to a slightly upregulated expression of these genes. Thus it is implied that the blocking of CD34^+^ cell differentiation in the SC cocktail group may result from the presence of small molecules. It is our plan to perform further investigation for the small-molecule combination using Wnt inhibitors to reveal its exact role on cell differentiation blocking. To sum up, these data demonstrated that the presence of the small-molecule combination can effectively inhibit the prodifferentiation effects of the cytokines without affecting the ability of the cytokines to stimulate cell proliferation.

## Conclusion

In conclusion, we established an optimal small-molecule and cytokine cocktail which can remarkably preserve the characteristics of HSCs by enhancing their expansion while inhibiting their differentiation. Mechanistically, it appears that these small molecules may affect the ability of cytokines to enhance the Notch pathway for the preservation of HSC stemness and, meanwhile, inhibit the ability of cytokines to intermediately activate the Wnt pathway to block HSC differentiation.

## Additional files

Additional file 1: Table S1. Various small-molecule concentrations and group distribution. The detailed concentration of single small-molecule screening for their individual effect on PB-CD34^+^ cell expansion. (xls 10.4 kb) (XLSX 10 kb)

Additional file 2: Table S2. Optimal small-molecule combination screening. (A) Selection of small-molecule combinations. (B) Concentration rearrangement of VPA at constant SR1 with the presence of three cytokines. (C) Concentration rearrangement of C433 at constant SR1 with the presence of three cytokines. (D) C433 minimization to determine the final optimal combination. (xls 11.3 kb) (XLSX 11 kb)

Additional file 3: Table S3. Cytokine combination selection. The detailed composition of different cytokine combinations. (xls 10.1 kb) (XLSX 10 kb)

Additional file 4: Table S4. Primer sequences for RT-PCR and qPCR. The detailed primers used for the detection of HSC specific gene expression and signaling pathway key and target gene expression. (xls 11.5 kb) (XLSX 11 kb)

Additional file 5: Figure S1. Cell viability and apoptosis of single small molecule selection. (A) Cell viability of three small molecules with various concentrations; 5000 cells were used as the initial number for each group and the absorbance was acquired on day 7 (mean ± SD, *n* = 3, *p < 0.05, **p < 0.01). (B) Apoptosis analysis of high dose group for each small molecule. The initial culture was started with 1 × 10^5^ cells and the data were obtained by FACS and analyzed by Flowjo software on day 7 (*n* = 3). (tif 8.73 MB) (PDF 1284 kb)

Additional file 6: Figure S2. Morphology of small-molecule combination screening. Photos were captured on day 7 (20× objective, scale bar = 50 μm). Vehicle was composed of DMSO. NC represents negative control with cytokines only. (PDF 2480 kb)

## Abbreviations

AHR: Aryl hydrocarbon receptor
APC: Allophycocyanin
BFU-E: Burst-forming unit-erythroid
C433/C633: CAY10433/CAY10633
CFU: Colony-forming unit
CFU-GEMM: Colony-forming unit-granulocyte, erythrocyte, macrophage, megakaryocyte
CFU-GM: Colony-forming unit-granulocyte, macrophage
CFU-Mk: Colony-forming unit-megakaryocyte
DMSO: Dimethyl sulfoxide
FACS: Fluorescence activated cell sorting
FITC: Fluorescein isothiocyanate
Flt3-L: Fms-related tyrosine kinase 3 ligand
HDACi: Histone deacetylase inhibitor
HSC: Hematopoietic stem cell
IL: Interleukin
NOD/SCID: Nonobese diabetic/severe combined immunodeficiency
PB: Peripheral blood
PBS: Phosphate-buffered saline
PCR: Polymerase chain reaction
PE: Phycoerythrin
qPCR: Quantitative polymerase chain reaction
RT-PCR: Reverse-transcription polymerase chain reaction
SCF: Stem cell factor
SCR: Scriptaid
SR1: Stemregenin1
SRC: SCID-repopulating cell
TPO: Thrombopoietin
UCB: Umbilical cord blood
VPA: Valproic acid

## References

[1] Walasek MA, van Os R, de Haan G (2012). Hematopoietic stem cell expansion: challenges and opportunities. Ann N Y Acad Sci.

[2] Shizuru JA, Negrin RS, Weissman IL (2005). Hematopoietic stem and progenitor cells: clinical and preclinical regeneration of the hematolymphoid system. Annu Rev Med.

[3] Daniel MG, Pereira CF, Lemischka IR, Moore KA (2016). Making a hematopoietic stem cell. Trends Cell Biol.

[4] Zhang C, Lodish HF (2008). Cytokines regulating hematopoietic stem cell function. Curr Opin Hematol.

[5] Flores-Guzman P, Fernandez-Sanchez V, Mayani H (2013). Concise review: ex vivo expansion of cord blood-derived hematopoietic stem and progenitor cells: basic principles, experimental approaches, and impact in regenerative medicine. Stem Cells Transl Med.

[6] Seita J, Weissman IL (2010). Hematopoietic stem cell: self-renewal versus differentiation. Wiley Interdiscip Rev Syst Biol Med.

[7] McNiece I, Harrington J, Turney J, Kellner J, Shpall EJ (2004). Ex vivo expansion of cord blood mononuclear cells on mesenchymal stem cells. Cytotherapy.

[8] Krosl J, Austin P, Beslu N, Kroon E, Humphries RK, Sauvageau G (2003). In vitro expansion of hematopoietic stem cells by recombinant TAT-HOXB4 protein. Nat Med.

[9] Rizo A, Dontje B, Vellenga E, de Haan G, Schuringa JJ (2008). Long-term maintenance of human hematopoietic stem/progenitor cells by expression of BMI1. Blood.

[10] Shen B, Zhang Y, Dai W, Ma Y, Jiang Y (2016). Ex-vivo expansion of nonhuman primate CD34+ cells by stem cell factor Sall4B. Stem Cell Res Ther.

[11] de Lima M, McMannis J, Gee A, Komanduri K, Couriel D, Andersson BS (2008). Transplantation of ex vivo expanded cord blood cells using the copper chelator tetraethylenepentamine: a phase I/II clinical trial. Bone Marrow Transplant.

[12] Peled T, Shoham H, Aschengrau D, Yackoubov D, Frei G, Rosenheimer GN (2012). Nicotinamide, a SIRT1 inhibitor, inhibits differentiation and facilitates expansion of hematopoietic progenitor cells with enhanced bone marrow homing and engraftment. Exp Hematol.

[13] Nishino T, Miyaji K, Ishiwata N, Arai K, Yui M, Asai Y (2009). Ex vivo expansion of human hematopoietic stem cells by a small-molecule agonist of c-MPL. Exp Hematol.

[14] Boitano AE, Wang J, Romeo R (2010). Aryl hydrocarbon receptor antagonists promote the expansion of human hematopoietic stem cells. Science.

[15] Hagedorn EJ, Durand EM, Fast EM, Zon LI (2014). Getting more for your marrow: boosting hematopoietic stem cell numbers with PGE2. Exp Cell Res.

[16] Chaurasia P, Gajzer DC, Schaniel C, D'Souza S, Hoffman R (2014). Epigenetic reprogramming induces the expansion of cord blood stem cells. J Clin Invest.

[17] Fares I, Chagraoui J, Gareau Y, Gingras S, Ruel R, Mayotte N (2014). Pyrimidoindole derivatives are agonists of human hematopoietic stem cell self-renewal. Science.

[18] Dahlberg A, Delaney C, Bernstein ID (2011). Ex vivo expansion of human hematopoietic stem and progenitor cells. Blood.

[19] de Lima M, McNiece I, Robinson SN, Munsell M, Eapen M, Horowitz M (2012). Cord-blood engraftment with ex vivo mesenchymal-cell coculture. N Engl J Med.

[20] Delaney C, Heimfeld S, Brashem-Stein C, Voorhies H, Manger RL, Bernstein ID (2010). Notch-mediated expansion of human cord blood progenitor cells capable of rapid myeloid reconstitution. Nat Med.

[21] Aggarwal R, Lu J, Pompili VJ, Das H (2012). Hematopoietic stem cells: transcriptional regulation, ex vivo expansion and clinical application. Curr Mol Med.

[22] Guan X, Qin M, Zhang Y, Wang Y, Shen B, Ren Z (2016). Safety and efficacy of megakaryocytes induced from hematopoietic stem cells in murine and nonhuman primate models. Stem Cells Transl Med.

[23] Liu B, Ohishi K, Yamamura K (2010). A potential activity of valproic acid in the stimulation of interleukin-3-mediated megakaryopoiesis and erythropoiesis. Exp Hematol.

[24] Cairo M, Wagner J (1997). Placental and/or umbilical cord blood: an alternative source of hematopoietic stem cells for transplantation. Blood.

[25] Almici C, Carlo-Stella C, Wagner JE, Rizzoli V (1995). Umbilical cord blood as a source of hematopoietic stem cells: from research to clinical application. Haematologica.

[26] La Motte-Mohs RN, Herer E, Zuniga-Pflucker JC (2005). Induction of T-cell development from human cord blood hematopoietic stem cells by Delta-like 1 in vitro. Blood.

[27] Qin M, Guan X, Wang H (2017). Ex vivo approch for inducing endothelial progenitor cells derived from umbilical cord blood CD34+ cells. Stem Cell Res Ther.

[28] Park IK, Qian D, Kiel M (2003). Bmi-1 is required for maintenance of adult self-renewing haematopoietic stem cells. Nature.

[29] Karpova D, Bonig H (2015). Concise review: CXCR4/CXCL12 signaling in immature hematopoiesis—lessons from pharmacological and genetic models. Stem Cells.

[30] Benveniste P, Serra P, Dervovic D (2014). Notch signals are required for in vitro but not in vivo maintenance of human hematopoietic stem cells and delay the appearance of multipotent progenitors. Blood.

[31] Bigas A, D'Altri T, Espinosa L (2012). The Notch pathway in hematopoietic stem cells. Curr Top Microbiol Immunol.

[32] Duncan AW, Rattis FM, DiMascio LN (2005). Integration of Notch and Wnt signaling in hematopoietic stem cell maintenance. Nat Immunol.

[33] Famili F, Brugman MH, Taskesen E, Naber BEA, Fodde R, Staal FJT (2016). High levels of canonical Wnt signaling lead to loss of stemness and increased differentiation in hematopoietic stem cells. Stem Cell Rep.

[34] Luis TC, Naber BA, Roozen PP (2011). Canonical wnt signaling regulates hematopoiesis in a dosage-dependent fashion. Cell Stem Cell.

[35] Langle D, Halver J, Rathmer B, Willems E, Schade D (2014). Small molecules targeting in vivo tissue regeneration. ACS Chem Biol.

[36] Lyssiotis CA, Lairson LL, Boitano AE, Wurdak H, Zhu S, Schultz PG (2011). Chemical control of stem cell fate and developmental potential. Angew Chem Int Ed Engl.

[37] Xu Y, Shi Y, Ding S (2008). A chemical approach to stem-cell biology and regenerative medicine. Nature.

[38] Zhang Y, Li W, Laurent T, Ding S (2012). Small molecules, big roles—the chemical manipulation of stem cell fate and somatic cell reprogramming. J Cell Sci.

[39] Russell AJ (2013). Regenerative medicinal chemistry: the in situ control of stem cells. ACS Med Chem Lett.

[40] Milhem M, Mahmud N, Lavelle D (2004). Modification of hematopoietic stem cell fate by 5aza 20 deoxycytidine and trichostatin A. Blood.

[41] Bug G, Gül H, Schwarz K (2005). Valproic acid stimulates proliferation and self-renewal of hematopoietic stem cells. Cancer Res.

[42] Hirabayashi Y, Inoue T (2009). Aryl hydrocarbon receptor biology and xenobiotic responses in hematopoietic progenitor cells. Biochem Pharmacol.

[43] Singh KP, Casado FL, Opanashuk LA, Gasiewicz TA (2009). The aryl hydrocarbon receptor has a normal function in the regulation of hematopoietic and other stem/progenitor cell populations. Biochem Pharmacol.

[44] Zardo G, Cimino G, Nervi C (2008). Epigenetic plasticity of chromatin in embryonic and hematopoietic stem/progenitor cells: therapeutic potential of cell reprogramming. Leukemia.

[45] Cedar H, Bergman Y (2011). Epigenetics of haematopoietic cell development. Nat Rev Immunol.

[46] Oh IH, Humphries RK (2012). Concise review: multidimensional regulation of the hematopoietic stem cell state. Stem Cells.

[47] Miller CL, Eaves CJ (1997). Expansion in vitro of adult murine hematopoietic stem cells with transplantable lympho-myeloid reconstituting ability. Proc Natl Acad Sci U S A.

[48] Piacibello W, Sanavio F, Garetto L (1997). Extensive amplification and self-renewal of human primitive hematopoietic stem cells from cord blood. Blood.

[49] Gilmore GL, DePasquale DK, Lister J, Shadduck RK (2000). Ex vivo expansion of human umbilical cord blood and peripheral blood CD341 hematopoietic stem cells. Exp Hematol.

[50] Audet J, Miller CL, Eaves CJ, Piret JM (2002). Common and distinct features of cytokine effects on hematopoietic stem and progenitor cells revealed by dose-response surface analysis. Biotechnol Bioeng.

[51] Fan J, Ding X, Jiang Y (2012). A novel monoclonal antibody of human stem cell factor inhibits umbilical cord blood stem cell ex vivo expansion. J Hematol Oncol.

[52] Shen B, Jiang W, Fan J, Dai W, Ding X, Jiang Y (2015). Residues 39-56 of stem cell factor protein sequence are capable of stimulating the expansion of cord blood CD34+ cells. PLoS One.

[53] Eaves CJ (2015). Hematopoietic stem cells: concepts, definitions, and the new reality. Blood.

[54] Conneally E, Cashman J, Petzer A, Eaves C (1997). Expansion in vitro of transplantable human cord blood stem cells demonstrated using a quantitative assay of their lympho-myeloid repopulating activity in nonobese diabetic-scid/scid mice. Proc Natl Acad Sci U S A.

[55] Dick JE, Bhatia M, Gan O, Kapp U, Wang JC (1997). Assay of human stem cells by repopulation of NOD/SCID mice. Stem Cells.

[56] Piacibello W, Sanavio F, Severino A (1999). Engraftment in nonobese diabetic severe combined immunodeficient mice of human CD34(+) cord blood cells after ex vivo expansion: evidence for the amplification and self-renewal of repopulating stem cells. Blood.

[57] Campbell C, Risueno RM, Salati S, Guezguez B, Bhatia M (2008). Signal control of hematopoietic stem cell fate: Wnt, Notch, and Hedgehog as the usual suspects. Curr Opin Hematol.

[58] Pajcini KV, Speck NA, Pear WS (2011). Notch signaling in mammalian hematopoietic stem cells. Leukemia.

