# Correction to: A small-molecule/cytokine combination enhances hematopoietic stem cell proliferation via inhibition of cell differentiation

**DOI:** 10.1186/s13287-021-02535-y

**Published:** 2021-08-17

**Authors:** Lan Wang, Xin Guan, Huihui Wang, Bin Shen, Yu Zhang, Zhihua Ren, Yupo Ma, Xinxin Ding, Yongping Jiang

**Affiliations:** 1 Biopharmaceutical R&D Center, Chinese Academy of Medical Sciences & Peking Union Medical College, Suzhou, China; 2 Biopharmagen Corp, Suzhou, China; 3 Department of Pathology, The State University of New York at Stony Brook, Stony Brook, NY USA; 4 College of Nanoscale Science and Engineering, SUNY Polytechnic Institute, Albany, NY USA

## Correction to: Stem Cell Research & Therapy (2017) 8:169 https://doi.org/10.1186/s13287-017-0625-z

Following the publication of the original article [1], the authors identified an error in Fig. 4b. The authors noticed that the gel bands of Runx1 were accidentally used again as the ones of Bmi1 during the layout of figures. It has been corrected after they double checked the original data. The results and conclusion concluded in this paper are still valid.

**Fig. 4:**
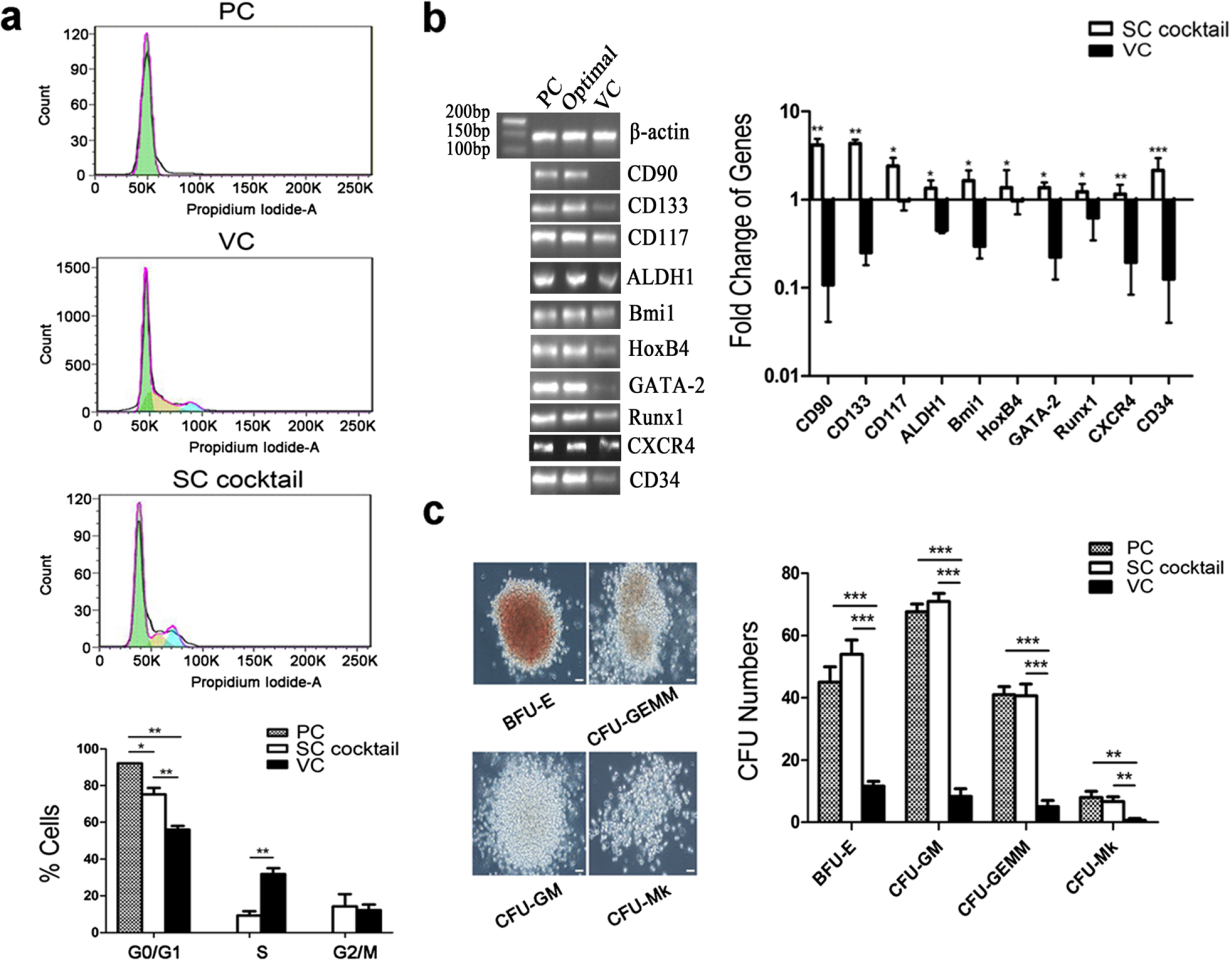
In vitro functional assessments of the SC cocktail-expanded CD34+ cells. **a** Cell cycle analysis. One of three representative experiments is shown, and the percentage of different phases is summarized in the histogram. **b** HSC-specific gene expression. Results of qualitative RNA-PCR are shown on the left for one representative of three independent RNA samples analyzed per group, and the results of quantitative PCR are shown in the right. **c** The morphology and numbers of colony-forming units (*CFUs*). Morphology (20× objective, scale bar = 50 μm) and colony numbers were recorded on day 14 after cell seeding. All data are shown as means ± SD, *n* = 3. *p < 0.05, **p < 0.01, ***p < 0.001. *BFU-E* burstforming unit-erythroid, *CFU-GEMM* CFU-granulocyte, erythrocyte, macrophage, megakaryocyte, *CFU-GM* CFU-granulocyte, macrophage, *CFU-Mk* CFU-megakaryocyte, *PC* uncultured cord blood HSCs, *VC* vehicle control
