# Supplementary figures and images for: A small-molecule/cytokine combination enhances hematopoietic stem cell proliferation via inhibition of cell differentiation
Source: Stem Cell Res Ther. 2017 Jul 18;8:169. doi: 10.1186/s13287-017-0625-z (PMC5516306; doi:10.1186/s13287-017-0625-z)

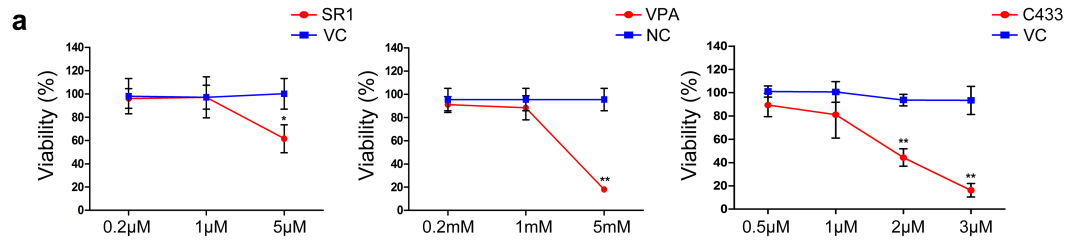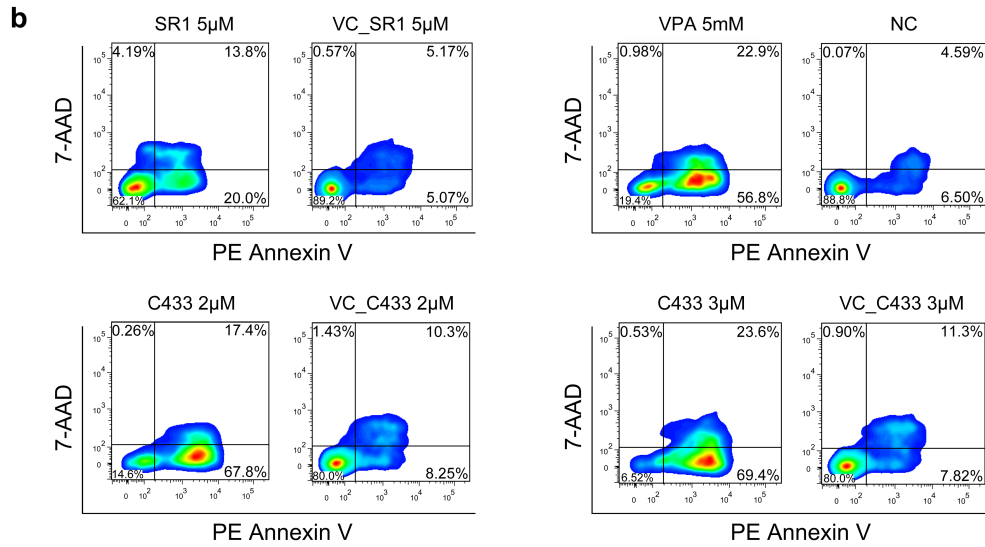

Supplement: Supplementary file 5 — Cell viability and apoptosis of single small molecule selection. (A) Cell viability of three small molecules with various concentrations; 5000 cells were used as the initial number for each group and the absorbance was acquired on day 7 (mean ± SD, n = 3, *p < 0.05, **p < 0.01). (B) Apoptosis analysis of high dose group for each small molecule. The initial culture was started with 1 × 10^5^ cells and the data were obtained by FACS and analyzed by Flowjo software on day 7 (n = 3). (tif 8.73 MB) (PDF 1284 kb) [file 13287_2017_625_MOESM5_ESM.pdf]

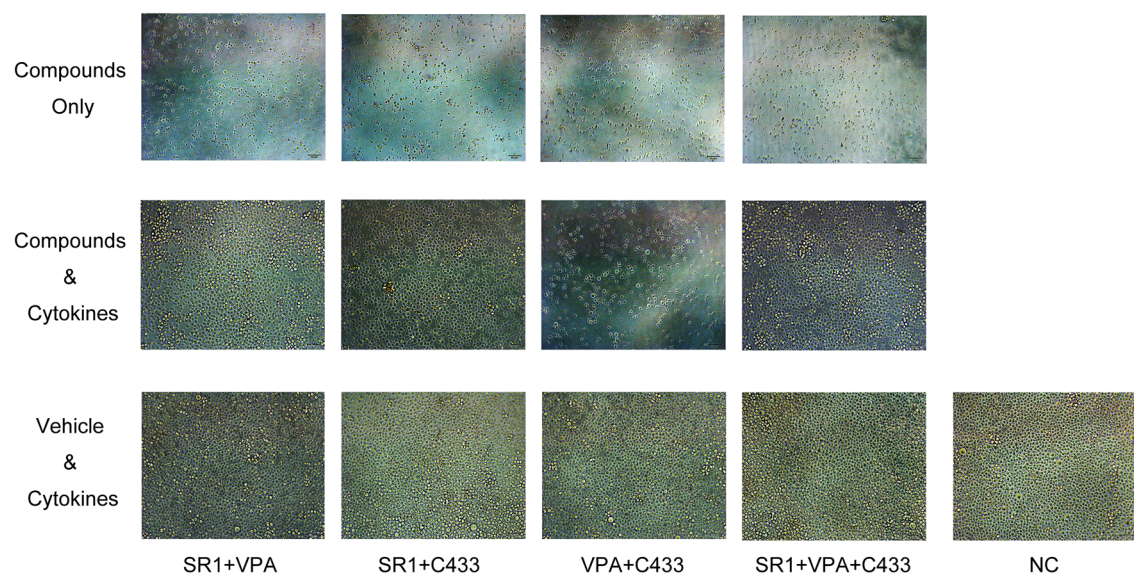

Supplement: Supplementary file 6 — Morphology of small-molecule combination screening. Photos were captured on day 7 (20× objective, scale bar = 50 μm). Vehicle was composed of DMSO. NC represents negative control with cytokines only. (PDF 2480 kb) [file 13287_2017_625_MOESM6_ESM.pdf]
